# Evidence, theory and context: using intervention mapping in the development of a community-based self-management program for chronic low back pain in a rural African primary care setting - the good back program

**DOI:** 10.1186/s12889-020-8392-7

**Published:** 2020-03-17

**Authors:** Chinonso N. Igwesi-Chidobe, Sheila Kitchen, Isaac O. Sorinola, Emma L. Godfrey

**Affiliations:** 1 Department of Medical Rehabilitation, Faculty of Health Sciences and Technology, College of Medicine, University of Nigeria (Enugu Campus), Nsukka, Nigeria; 2 Department of Physiotherapy, Faculty of Life Sciences and Medicine, School of Population Health Sciences, King’s College London, London, SE1 1UL UK; 3 Department of Psychology, Institute of Psychiatry, Psychology and Neuroscience, King’s College London, London, UK

**Keywords:** The Good Back Program, Intervention Mapping, Self-management program, Chronic low back pain, Complex interventions, Behavior change intervention, Rural Africa

## Abstract

**Background:**

Rural Nigeria has one of the greatest burdens of low back pain but there are no effective evidence-based interventions to manage it in this population. This paper presents the application of the intervention mapping (IM) approach in the development of a complex behavior change intervention – The Good Back program, aimed at reducing non-specific chronic low back pain (CLBP) disability in rural Nigeria.

**Methods:**

The first four steps of IM were applied. A critical review of the literature, 2 qualitative studies and a population-based cross-sectional study in rural Nigeria helped to identify two key program objectives in order to reduce CLBP disability in this population: reduce the impact of illness perceptions, fear avoidance beliefs, catastrophising, anxiety and depression by targeting maladaptive illness perceptions about CLBP; and facilitate the adoption of exercises and good posture to limit disability. A systematic review plus these studies, identified the personal and environmental determinants of the performance objectives including health literacy, self-awareness, self-efficacy, personal preference, health professional skills, health facility structure and family/community support. The theory, techniques and strategies for modifying personal and environmental determinants were also identified from these studies. Intervention components and materials were then produced for practical application. The initial developed intervention was described.

**Results:**

The feasibility and acceptability of the developed program was then tested using a small pragmatic non-randomised controlled study incorporating qualitative exit feedback interviews in a rural Nigerian primary health care centre. The program appeared feasible and acceptable when delivered by a highly trained physiotherapist. There were promising clinical outcomes in disability, pain intensity, illness perceptions, fear avoidance beliefs and pain medication use. Suggestions for program improvement included shorter but ongoing sessions, video demonstration of exercises/good posture, spacious exercise/demonstration rooms, and community legitimisation of exercise as treatment for back pain. Subsequent modifications to program content and delivery were then described. Theoretical modification included the addition of aspects of the social cognitive theory to the Leventhal’s self-regulatory model of illness cognitions.

**Conclusions:**

IM appears to be a suitable framework for designing complex behavior change interventions in rural Nigeria. The need for further testing of the intervention was highlighted.

## Background

Low back pain (LBP) is the leading cause of years lived with disability worldwide [1, 2]. The course of LBP is increasingly viewed as chronic, because recurrence of an acute episode of LBP is almost certain [3]. The disability associated with LBP has increased by more than 50% since 1990, particularly in low-income and middle-income countries [4]. The impact is likely to be more devastating in rural African and other low-income contexts where cultural beliefs, unsafe practices, adverse environmental conditions, combined with high levels of poverty and lack of health services may increase the risk and impact of LBP [5, 6]. Nigeria appears to have one of the greatest burdens of chronic low back pain (CLBP) in the world. This is particularly in rural Nigeria with a 1-year prevalence rate reaching up to 85%, a much higher figure than the 39% in urban Nigeria [7, 8]. Despite this significant burden and impact, there are no evidence-based interventions for the management of CLBP in rural Nigeria. Evidence-based clinical guidelines for CLBP recommend the adoption of a biopsychosocial framework incorporating self-management, exercises and psychological treatment such as cognitive behavioral therapy – CBT [9].

Frameworks for developing complex interventions often have limitations in terms of their applicability to any model or theory of behavior, acknowledgement of contextual factors, and translation of these into actual program materials and activities. Although, the behavior change wheel acknowledges contextual factors, it is specifically linked to theories of motivation [10]. The MINDSPACE framework emphasizes the influence of contextual factors on behavior but does not specify the need for theory in intervention development [11]. None of these frameworks provided concrete steps to follow in the translation of theory and environmental factors into program content. The Intervention Mapping (IM) [12] does not appear to have these limitations.

IM is compatible with any theory, and provides strategies for identifying the determinants of the desired behavior, and matching them with appropriate theory-based methods for change [12]. Another reason for the choice of the IM approach was because of its consistency with the Medical Research Council guidance [13]. IM may be useful in developing complex behavior change interventions in Africa since its social ecological paradigm acknowledges both personal and environmental factors as determinants of health. The IM approach was useful in developing a community-based intervention that aimed to increase consumption of a healthy balanced diet, increase physical activity, reduce sedentary behavior and promote reproductive health in adolescents in a rural South African community. While acknowledging multiple domains of influence: community, family and individual, IM enabled the tailoring of a western intervention to suit this rural African context [14]. The evaluation of an intervention developed using IM to target sexual and reproductive health in early adolescents in Tanzania and South Africa, enabled the modification of social cognitive models to fit an African context [15]. For instance, the intervention acknowledged that the influence of cognitions on behavior may be limited by contextual and social barriers to the desired behavior in Africa, e.g. availability of condoms for safe sexual practices.

The IM approach iteratively moves from problem identification to problem solving. It has 6 steps: needs assessment; detailed mapping of program objectives and their behavioral and environmental determinants; selecting theory and techniques/strategies to modify the determinants of behavior and the environment; producing intervention components and materials; implementation; and evaluation [12]. The completion of each step serves as a guide for the subsequent step. The first step is used to identify what needs to be changed and for whom. The second step involves the development of matrices of change objectives. The third step contains theory-based intervention methods for targeting the performance objectives and their determinants. Methods, materials and practical applications are integrated into an organized program in step four. Step five includes planning for intervention adoption, implementation and sustainability in real-life contexts. In the final step, plans for conducting efficacy and process evaluations are drawn up [12].

This paper will (1) describe the initial development of a self-management program aiming to reduce CLBP disability in rural Nigeria using the first four steps of the IM approach, (2) describe the initial implementation and feasibility testing, (3) describe the initial self-management program before feasibility testing, and (4) highlight the changes made to the program following feasibility testing to produce a definitive program. This paper is reported acknowledging the items in the template for intervention description and replication (TIDieR) checklist and guide [16].

## Methods

### Step one: needs assessment

**Fig. 1:**
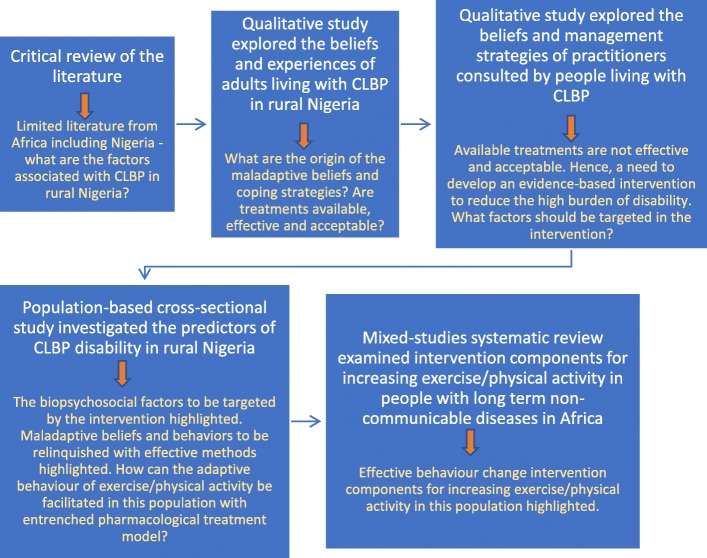
Order of studies conducted for initial intervention development

This involved analysing the problem of CLBP in rural Nigeria, its associated behavioral and environmental conditions, and the determinants of these conditions. Figure 1 below highlights the studies that informed initial intervention development. A thorough review of the literature showed that physical, psychological and social factors including somatisation, psychological distress, fear avoidance beliefs, catastrophising, illness perceptions, and coping strategies may be implicated in CLBP disability. Job-related psychosocial factors such as job satisfaction, number and extent of working hours, and work organizational factors were not associated with sick leave. Physical factors such as increased and prolonged trunk flexion and twisting, and spinal loading were more important in first onset LBP than in CLBP and the associated disability. However, most of the evidence were from high income countries [17].

Qualitative research is the best way to begin to study an area with little previous research. Therefore, the first study involved the qualitative exploration of the experiences of people living with CLBP in rural Nigeria. Thirty in-depth semi-structured face-to-face interviews were conducted with purposively selected participants until data saturation. Questions explored back pain beliefs, coping strategies and daily activities. Framework approach was used in the thematic analysis of transcripts. Findings highlighted maladaptive beliefs including the social construction of back pain as a disease of hard labour and deprivation which might be linked to fear avoidance beliefs, catastrophising and hopelessness; and spiritual causal beliefs or beliefs that CLBP was due to infection which led to coping strategies such as spiritual healing expectations, cure seeking and drug dependence resulting in high levels of emotional distress and disability amongst people living with CLBP [18]. In-depth semi-structured face-to-face interviews with twelve unconventional practitioners – patent medicine sellers (4), herbalists (3) and pastors (2) explored their beliefs about CLBP, management strategies, and perceived effectiveness of these strategies [19]. Thematic analysis using the framework approach was applied. Patent medicine sellers and herbalists had biomedical beliefs about CLBP and encouraged passivity and drug dependence in patients. Pastors adopted spiritual or biopsychosocial-spiritual management models and either encouraged spiritual CLBP causal beliefs and spiritual healing expectations; or patients’ resilience and acceptance [19]. These practitioners’ CLBP beliefs and management strategies appeared to reinforce maladaptive beliefs and coping strategies among the people living with CLBP hence contributing to the severe impact of CLBP in rural Nigeria. Therefore, the perceived biopsychosocial factors associated with the experience of CLBP from these studies were work-related biomechanical factors, illness perceptions, fear avoidance beliefs, pain intensity, anxiety, depression, catastrophising, coping and social support.

The generalisability of these findings and the individual contribution of the perceived biopsychosocial factors to CLBP disability in rural Nigeria remained unknown. Consequently, a population-based exploratory cross-sectional study of a representative sample of 200 people living with CLBP in 10 rural Nigerian communities investigated the biomechanical and psychosocial predictors of CLBP disability in rural Nigeria. Results showed that psychosocial factors were the most important predictors of CLBP disability and accounted for 62.5 and 49.1% of the variance in self-reported and performance-based disability, respectively. Illness perceptions (β = 0.289; *p* < 0.0005), pain intensity (β = 0.230; *p* < 0.0005), catastrophising (β = 0.210; *p* = 0.001), fear avoidance beliefs (β = 0.198; *p* = 0.001) and anxiety (β = 0.154; *p* = 0.023) were the significant predictors of self-reported disability. Illness perceptions (β = 0.366; *p* < 0.0005), social support (β = 0.290; *p* < 0.0005), fear avoidance beliefs (β = 0.189; *p* < 0.01) and female gender (β = 0.184; *p* < 0.01) were the significant predictors of performance-based disability. Notably, illness perceptions had the strongest associations with both self-reported and performance-based disability; and fear avoidance beliefs were also associated with both self-reported and performance-based disability [6]. The comparatively weaker association of anxiety and depression with CLBP disability may be due to unclear expression of emotional concepts in rural Nigeria [20].

The critical review of literature [17] also found high quality evidence that included several systematic reviews of randomised controlled trials conducted in high income countries showing that exercise therapy improved pain, disability and long-term function in people living with CLBP compared to usual care or non-active control groups. However, there was no clear evidence for the superiority of any type of exercise. Exercise of any type is thought to decrease fear-avoidance behaviour and facilitate functional improvements, despite ongoing pain. Another reason for the non-specific effects of exercise could be due to central nervous system effects, as exercise increases pain thresholds due to the release of endogenous opioids, growth factors, and other strong inhibitory mechanisms via descending inhibition driven by the central nervous system [17]. This is important as endogenous inhibition of pain during exercise is normal in people with CLBP. Strengthening activities incorporating motor control and flexibility exercises were common in effective exercise interventions. Supervision improved exercise adherence and was a common feature of the trials that maintained positive results at short and long-term follow-up [17]. Exercise improves back flexibility, strength and performance of endurance activities, and reduces pain intensity and disability through desensitisation of fears, and alteration of pain beliefs and attitudes. Unimodal exercise therapy is less effective than biopsychosocial multidisciplinary treatment incorporating exercise, especially in relation to long-term work outcomes [17]. Back schools are educational programs about back care, posture, body mechanics and back exercises, and have been used in CLBP management. Numerous systematic reviews demonstrate that comprehensive back schools that acknowledge physical, psychological and social factors may be superior to less comprehensive ones in reducing pain and CLBP disability. Hence, comprehensive programs that combined psychological strategies with exercises and postural training may be effective in reducing CLBP disability in rural Nigeria where people are mainly involved in physically demanding manual occupations [21–23].

Therefore, the overall aim of the program was to reduce CLBP disability by achieving two key program objectives:

1. Reduce the impact of illness perceptions, fear avoidance beliefs, catastrophising, anxiety and depression by targeting maladaptive illness perceptions about CLBP.
2. Facilitate the adoption of exercises and good postural hygiene to limit disability

### Step two: detailed mapping of performance objectives with their personal and environmental determinants to form a matrix for each program objective

Tables 1 and 2 below illustrate the two matrices developed for the two program objectives identified in step one.

**Table 1:** Matrix of change for programme objective 1 - Reduce the impact of illness perceptions, fear avoidance beliefs, catastrophising, anxiety and depression by targeting maladaptive illness perceptions about CLBP

	**Personal determinants**			
**Performance objective**	Health literacy	Self-awareness	Self-efficacy	Personal preference
Formulate alternative adaptive illness perceptions about CLBP	Increase knowledge about CLBP and its causes	Awareness of existing maladaptive illness perceptions that result in maladaptive coping strategies (drug dependence and cure seeking)	Ability to challenge maladaptive illness perceptions and adopt adaptive ones	Ability to choose adaptive illness perceptions that will reduce maladaptive coping strategies (drug dependence and cure seeking)
	**External/environmental determinants**			
	Health professional skills		Health facility structure	
	Health professional has the skills to facilitate participants’ formulation of alternative adaptive illness perceptions about CLBP by taking them through the required steps: provide information, create awareness of maladaptive illness perceptions and support the challenge of these perceptions. This means providing alternative explanations where appropriate which may include challenging pessimistic illness cognitions, and using problem-focussed coping behaviours to manage stressors and improve any distress.		Health facility is accessible and adequate to support free discussion by participants and health professional

**Table 2:** Matrix of change for programme objective 2 - Facilitate adoption of specific exercises and correct posture

	**Personal determinants**			
**Performance objective**	Health literacy	Self-awareness	Self-efficacy	Personal preference
Take up exercises and good posture as self-management strategies for CLBP	Increase knowledge of exercises and good posture as effective long term management options for CLBP	Awareness of current negative beliefs about exercises and good posture	Learn specific exercises and good posture, and have the ability to incorporate them into daily life	Ability to decide which exercises to perform at different times in daily life
	**External/environmental determinants**			
	Health professional skills	Health facility structure		Family support
	Health professional has the skills to teach participants specific exercises and good posture, and support their adoption in daily life	Health facility structure is accessible and supports performance of group exercise and postural training sessions		Family members support adoption of exercises and good posture in daily life

Each key program objective identified in the needs assessment is broken down into performance objectives. Behavioral determinants are generic aggregates of beliefs, that are specific to a behavior, population, and context while personal determinants were the generic modifiable psychological variables that are assumed to be causal antecedents of behavior [24]. External determinants are factors external to the individual that may influence behavior. Adopting the social ecological paradigm, which acknowledges both personal and environmental determinants of behaviour, the lead author independently identified the personal and external determinants of the performance objectives from the results of the needs assessment studies. This was independently appraised by each member of the multidisciplinary research team, comprising clinical and academic physiotherapists and health psychologists. Consensus agreement in the determinants of each performance objective was achieved through group discussion of the multidisciplinary team. The determinants were specified for each of the performance objectives to form a matrix for each program objective.

We illustrate how the determinants of behaviour were identified from the needs assessment studies. The results of the qualitative studies showed that people in rural Nigeria did not have appropriate information about CLBP which appeared to foster various maladaptive beliefs which resulted in maladaptive coping strategies. For instance, people’s belief that CLBP was a disease of hard labour and deprivation made them feel that CLBP was inevitable to them as rural dwellers and this inhibited adaptive behavioural modification and increased the feelings of hopelessness, helplessness, anxiety and depression, leading to pain medication dependence. Participants’ beliefs that CLBP was a sign of infection or ageing led them to believe it caused their infertility or that they were ageing prematurely which increased catastrophising and cure seeking. Their beliefs that CLBP was due to spiritual attack led to attempts to receive spiritual healing through unconventional practitioners [18, 19]. The above factors are positively associated with CLBP disability in rural Nigeria [(illness perceptions (β = 0.289; *p* < 0.0005); pain intensity (β = 0.230; *p* < 0.0005); catastrophising (β = 0.210; *p* = 0.001); fear avoidance beliefs (β = 0.198; *p* = 0.001); anxiety (β = 0.154; *p* = 0.023); perceived spiritual causal factors (*p* = 0.039)] [6]. It was therefore necessary that health literacy should be targeted as a personal determinant of maladaptive beliefs, maladaptive coping strategies and exercise behaviour. Self-awareness, self-efficacy and personal preference were included as personal determinants of all the performance objectives as they are self-regulation constructs. Self-regulation constructs were identified from the studies conducted during the development of this program. The critical review of literature [17] found that self-regulation constructs are necessary for bridging intention-behavior gap [25]; and have been found to be useful part of behavior change interventions in rural Africa [14]. Importantly, the self-regulatory model of illness perceptions sufficiently explained the link between CLBP beliefs, emotional responses, coping strategies; and CLBP disability in rural Nigeria [6, 18]. Furthermore, interventions containing self-regulation constructs were more likely to improve physical activity behaviour of adults living with chronic non-communicable diseases in Africa than interventions that do not contain them [26].

One of the program objectives was to facilitate the adoption of exercises. However, the results from the qualitative studies showed that exercise was not regarded as legitimate treatment in this population. Therefore, a systematic review investigated the behavior change intervention components for improving acceptability and exercise/physical activity behaviour among African patients with chronic non-communicable diseases living in Africa. Clinical and qualitative studies that examined the effects and patients’ experiences of physical activity interventions for chronic NCDs in Africa were obtained by searching eight bibliographic databases and grey literature until April 2017 and subsequently reviewed. Results showed that behaviour-change techniques that improve chronic disease knowledge, address environmental barriers and stimulate/support physical activity were important to patients. Procedure-related components—health professional training and adequate health facilities were important to patients to support physical activity programs and behavior [26]. To improve chronic disease knowledge, organized, simple and comprehensive structured educational programs, covering all aspects of the patients’ chronic condition including management strategies were important to patients. Recapping previous educational sessions enhanced recall. Communication styles incorporating motivational interviewing principles such as empathy, liveliness, inclusiveness and collaborative group sessions enhanced understanding. Self-help educational materials, such as handouts, flip charts, booklets and posters, enhanced understanding and recall, and made the sessions more interesting. Health education delivered within mainstream healthcare settings bestowed value and legitimacy to self-management recommendations [26]. To facilitate physical and social environment that supports physical activity, unsafe environments, poor timing of physical activity, family problems and stressful work conditions should be addressed. Social support facilitated through group sessions should be encouraged as it appeared to mitigate stressors. To stimulate physical activity behaviour change, motivational interviewing techniques, such as eliciting talk about change, using enthusiastic communication styles and encouraging group solutions were useful. Emphasizing chronic disease complications appeared to further increase the motivation to improve physical activity; and actual performance of the desired physical activity seemed to increase physical activity through increasing self-efficacy. For the procedure-related components, adequate training of health professionals in the biopsychosocial model of chronic disease management was important as health professionals operating within a biomedical health care system were more likely to be impatient with patients, damaging the patient–health professional relationship. Limiting the targeted physical activity behaviour change were inadequate venues or programs that were not integrated into routine care. This resulted to conflicting opinions about who should lead interventions, and timing problems. Patients wanted ongoing programs with the possibility of repeat sessions, with specific dates and times that aligned with other hospital appointments/daily activities, and programs delivered by specifically trained health professionals [26].

Based on these facts above, the personal determinants for the performance objective - formulating adaptive alternative illness perceptions about CLBP were depicted in Table 1. External determinants for formulating adaptive alternative illness perceptions about CLBP were also highlighted in Table 1.

As detailed above, the determinants for exercise and good postural hygiene were based on the findings of the first qualitative study suggesting that exercise was not viewed as legitimate treatment in this population with an entrenched pharmacological treatment model [18] and the systematic review which highlighted the intervention components and the environmental determinants which might be relevant for improving exercise behavior in this population [26]. Based on the findings above, the personal determinants for exercising and postural awareness for self-managing CLBP were highlighted in Table 2. The external determinants for exercising and adopting good postural hygiene for self-managing CLBP were also detailed in Table 2. Family support was added as an external determinant of exercises and good postural hygiene because the systematic review also suggested that restructuring the social environment– such as the family may be important for physical activity related behavior change in Africa (Table 2).

These matrices of change provided a map that facilitated the identification of specific intervention components, methods, strategies and materials.

### Step three: selecting theory, techniques and strategies to modify the determinants of behavior and the environment

The critical review of literature [17], results of the two qualitative studies [18, 19], and a population-based cross-sectional study [6] suggested that the Leventhal’s self-regulatory model of illness cognitions (SRM) [27] may be relevant in understanding health behavior in Nigeria. Notably, illness perceptions were the strongest predictors of self-reported and performance-based CLBP disability in rural Nigeria [6].

The SRM proposes that symptoms generate both cognitive and emotional representations of a potential illness through three stages: interpretation, coping and appraisal. These representations then influence which behaviors are adopted for coping, while the efficacy of these behaviors is appraised in the final stage to determine whether or not to continue with the coping strategies [27]. SRM has explained CLBP outcomes in high income countries [28, 29] but there is a dearth of theory-driven health behavior change interventions in Nigeria.

Consequently, theory-based methods aligned with the SRM were identified from the taxonomy of behavior change methods when using the intervention mapping approach [24]. The behavior change techniques used to deliver these methods were labelled according to the behavior change taxonomy of 93 hierarchically clustered techniques [30]. Cognitive behavioral therapy (CBT) was seen as an appropriate theory-based method for challenging illness perceptions, consistent with the SRM [31–33]. CBT explains that the way in which a person thinks about their problem will produce emotions, including associated physical sensations, which then drive behavior. The behavior can subsequently maintain the thoughts/beliefs, thus retaining the impact of the health condition in a vicious cycle [34]. CBT provides a toolkit of interventions to target cognitions/beliefs, emotions and behaviors interactively, and embodies a range of interventions that aim to change behavior directly using models of learning, and indirectly by changing beliefs [35]. CBT has been useful in group-based [36, 37] and individual-based [38, 39] interventions. However, CBT alone has been shown to be ill equipped for managing individuals with compromised cognitive function, or low motivation for behavior change in western settings [40, 41]. In rural Nigeria, high illiteracy rates and a biomedical CLBP model may create ambivalence to behavior change.

It was necessary to explore the factors that might improve acceptability of exercises for managing CLBP. From the systematic review, motivational interviewing was found useful for communicating health information and facilitating physical activity behavior change in people with chronic non-communicable conditions in Africa, although no study on chronic pain or CBT was identified [26].

Motivational interviewing is defined as a collaborative, goal-oriented style of communication that pays particular attention to the language of change to strengthen patients’ motivation and commitment to changing a particular behavior by exploring their reasons for change within an atmosphere of acceptance and compassion. Motivational interviewing has four fundamental processes: engaging, guiding, evoking and planning [42]. Similar to the CBT, it is a behavior change method that also aligns with the SRM. However, unlike CBT, it may also be useful in individuals with compromised cognitive function or low motivation for behavior change [40, 41].

Therefore, integrating techniques from CBT and motivational interviewing to equip individuals with practical steps to enable behavior change appears to be a logical approach in rural Nigeria. Separating treatment phases so that the essential principles of each approach are not compromised [40], may be one way of responding to the call for research improving CBT participation and efficacy in individuals with low literacy levels [43]. This was acknowledged in the development of the Good Back program by having treatment phases that were either informed by CBT or motivational interviewing as detailed in the description of treatment phases in the results section.

### Step four: producing intervention components and materials for practical application

Detailed program components and materials are illustrated in Table 3 below. The lead author identified the intervention components to facilitate the targeted behavior change, and the procedures for the delivery of those BCTs and this was refined via discussion with the multidisciplinary research team. Intervention components for achieving specified program objectives were informed by the results of completed studies. These studies helped identify the relevant themes to explore in the program. The program themes addressed relevant biopsychosocial factors including illness perceptions, fear avoidance beliefs, catastrophising, anxiety, depression, occupational biomechanical factors and coping strategies.

**Table 3:** Intervention components

**Intervention components: BCTs and procedures**								
**Intervention phases/materials**	**Theory-based behaviour change methods**	**Delivery informed by**	**Behavior Change Techniques (BCTs)- labelled from** the Behavior Change Technique Taxonomy (v1) of 93 hierarchically clustered techniques	**Intervention format** Face to face, group exercise sessions, group + individual discussion sessions	**Intervention provider** Physiotherapist trained in behaviour change techniques	**Duration of intervention** 6 weeks	**Frequency of intervention** One per week	**Context within which intervention was delivered** Rural primary health care centre
Education/ Patient and Physiotherapist booklets	MI	MI	*Shaping knowledge* (instruction on how to perform a behaviour, re-attribution), *Natural consequences* (Information about health consequences)					
Mapping of existing illness perceptions/ Physiotherapist booklet	Improving physical and emotional states	CBT	*Natural consequences* (Monitoring of emotional consequences - by self Monitoring of functional consequences^a^ - by self)					
Challenging maladaptive illness perceptions/ Physiotherapist booklet	Improving physical and emotional states	CBT	*Natural consequences* (Information about emotional consequences Information about functional consequences^a^					
Formulation of alternative illness perceptions and associated behaviours/ Patient and Physiotherapist booklets	Improving physical and emotional states	CBT	*Natural consequences* (Information about emotional consequences) *Self-belief* (Verbal persuasion about capability), *Natural consequences* (Information about functional consequences)^a^					
Practising the alternative (desired) behaviour (exercises and good posture) in a supervised session/ Patient and Physiotherapist booklets	Guided practice, Goal setting	MI	*Shaping Knowledge* (Instruction on how to perform a behaviour), *Self-belief* (Verbal persuasion about capability), *Repetition and substitution* (Behavioural practice/rehearsal, Habit formation)					
Testing of alternative illness perceptions and associated behaviours/ Patient booklet	Guided practice, Self-monitoring of behaviour, Set graded tasks, Planning coping responses	CBT	*Shaping knowledge* behavioural experiments) *Social support* Social support (unspecified), *Antecedents* (Restructuring the social environment, Restructuring the physical environment) *Feedback and monitoring* (Self-monitoring of behaviour, Self-monitoring of outcome(s) of behaviour)					

*MI* Motivational interviewing, *CBT* Cognitive behavioural therapy

^a^absent in the 93 BCT

Intervention components were the behavior change techniques (BCTs) used to facilitate behavior change, and the procedures for the delivery of those BCTs [30]. The procedures include intervention format, intervention provider, intervention duration, intervention frequency and the context within which the intervention was delivered. The materials included the physiotherapist and patient booklets (Table 3).

The intervention format selected was face-to-face based on the socio-cultural and socio-economic context of rural Nigeria. Previous public health projects in this context suggest that they were unlikely to regard programs that are not face to face as legitimate. Moreover, some of the rural dwellers especially the elderly, lacked access to internet and telephone. It was also important that the physiotherapist delivering the intervention was trained in behavior change methods, as evidenced by the systematic review, which suggested that a biomedically oriented health practitioner may inhibit behavior change [26].

The program aligned with the Stanford self-management support approach: 6 weeks’ intervention, use of self-regulatory approaches, group-based (ten to fifteen participants per group), peer influence [44]. Individual discussion sessions and delivering the intervention within primary care were additional program procedures aimed at overcoming the problems of confidentiality and accessibility reported in the Stanford model.

### Description of initial self-management program before feasibility testing

#### Brief name

The Good Back program – promoting intervention goals and ownership by the rural dwellers.

#### Elements essential to the intervention

CBT to challenge maladaptive CLBP cognitions and associated behaviors; and motivational interviewing to facilitate the adoption of exercises and good postural hygiene in daily life (adaptive behaviours).

The exercises were adapted to suit the rural Nigerian context by including simple exercises that do not require the use of equipment; and including picture models that look similar to adult rural Nigerian dwellers in the program booklets. The exercises included aerobic, strengthening, neuromuscular, flexibility, and relaxation exercises; and incorporated both the principles of exercise physiology and behavior change principles. As no evidence supports any specific exercise progression for the self-management of CLBP, participants are encouraged to progressively increase their daily exercises based on individual tolerance levels. Each program session includes warm up exercises, back specific exercises, and cool down exercises. Participants are educated about exercise-induced pain secondary to delayed muscle soreness during the educational sessions to avoid fears about reinjury which could undermine participation. Good postural hygiene was demonstrated with culturally relevant daily/work activities.

#### Procedures, activities, and processes used in the intervention

This is a six-week group self-management program that incorporates individual exercise sessions with discussion sessions; administered once weekly.

The program was designed to adapt to people’s socio-cultural contexts, as participants are encouraged to discuss their life’s goals.

The six program sessions were based on six themes as illustrated in Table 4 below. The lead author initially developed the educational themes from the results of all the studies conducted (Fig. 1). For instance, the content and specific expression of the maladaptive beliefs and coping strategies, which are predictors of CLBP disability in the population-based cross-sectional study, were identified from the two qualitative studies, and used in the development of the themes that challenged the maladaptive beliefs and coping strategies. The critical literature review and systematic review informed the educational themes about CLBP management. The multidisciplinary team members independently reviewed initial themes and made comments. A final version was developed following two discussion sessions.

**Table 4:** The six themes of the six programme sessions

Theme 1 (Session 1)	Challenging the biomechanical understanding of non-specific chronic low back pain (CLBP). Exercises and postural training are noted as important tools in managing CLBP. The educational aspect of the session covers spinal anatomy and physiology, epidemiology of nonspecific low back pain, exercises, and postural education. The theme is related to participants’ understanding of CLBP as a disease of hard labour, deprivation and rural habitation, specific environmental factors and rural health care facilities. Discussion involves explanation of CLBP as ubiquitous regardless of the level of exposure to biomechanical factors; exploration of good attributes of rural habitation and identifying participants’ ability to influence their own environment, example working from a table rather than the ground.
Theme 2 (Session 2)	Challenging the infective-degenerative explanation of CLBP. The educational aspect of the session covers the epidemiology of specific back pain. This is related to participants’ infective-degenerative understanding of CLBP with explanation that most cases of CLBP are not due to infection. The differences between the symptoms and treatment of specific back pain and non-specific back pain are explored with the application of analogies.
Theme 3 (Session 3)	Challenging negative beliefs and thoughts about back pain including sexuality, fertility, occupational activities and spiritual explanations of CLBP. The educational aspect of the session covers challenging thoughts about back pain. Participants are educated and empowered to challenge and control their thoughts/beliefs and the impact they have on their back pain. Occupational activities and the spiritual understanding of CLBP are explored in group sessions. The utility of spiritual causal explanations of CLBP is compared with spirituality leading to pain acceptance, relinquishment of the sick role and social support. The effect of CLBP on occupational activities and the impact on gender roles are explored with participants. For participants who have questions about the role of CLBP on sexuality and fertility, individual sessions may be used to explore gender roles in terms of sexuality and fertility due to the sensitive nature of this topic. The importance of communication for sexuality should be discussed, and the lack of association between CLBP and fertility in women can be explained. People with fertility problems should be encouraged to visit the hospital and seek medical advice.
Theme 4 (Session 4)	Managing exercise, pacing, goal setting and relaxation, the use of exercise both as a preventive and therapeutic tool in CLBP, and the role of drugs in CLBP. The educational aspect of the session covers managing exercise, pacing, goal setting and relaxation. Participants’ understanding of exercise either as a preventive or therapeutic strategy is explored. The explanation emphasizes how exercise fulfils both roles and the different types of exercises are discussed. This is related to the use of drugs in CLBP with an explanation that drugs are only meant to be used temporarily for short-term relief of symptoms and that no drug cures CLBP. Explanation about the dangers of drug dependence such as toxicity and inhibition of adaptive behaviour change are provided.
Theme five (Session 5)	Biopsychosocial model of CLBP and the chronic disease model. The educational aspect of the session covers chronic pain and chronic disease such as hypertension and diabetes. The differences between acute and chronic conditions and their management are explored with participants. Participants are facilitated in developing an understanding of the contribution of psychosocial factors to the impact of not only CLBP but also the common chronic conditions in rural Nigeria including hypertension and diabetes. Participants are supported in identifying relevant psychosocial factors influencing the impact of CLBP in their lives and how they can control them.
Theme six (Session 6)	Self-management of CLBP, help seeking, managing and coping with flare ups. The educational aspect of the session covers managing and coping with flare ups, relaxation, help seeking and self-management. CLBP is compared with other chronic conditions such as diabetes and hypertension. The difference between the conditions in relation to drug use is explored. Participants are helped to explore how people managed acute conditions such as malaria, and how this is different from how people managed chronic conditions such as diabetes. Participants are then encouraged to explore the similarity and differences between the management of CLBP and other chronic conditions such as hypertension and diabetes.

Each of the six program sessions is divided into six phases as illustrated in Table 5 below. The number of phases and their contents were informed by the critical literature review, the previous studies including the systematic review. For instance, the lead author identified the efficacy of using motivational interviewing principles for the educational and exercise performance phases of the program from the systematic review, whereas the procedures for targeting illness perceptions were informed by a randomised controlled trial of the cognitive treatment of illness perceptions in patients with CLBP [29]. The treatment phases were separated by using either motivational interviewing or CBT principles. For instance, phase 1, which is the educational phase of the intervention uses motivational interviewing principles to ensure that health information (including information about CLBP) is adequately understood by this population with limited literacy; whilst phase 5 which involves the practice of the desired behaviour is targeted at facilitating the adoption of exercise and good postural hygiene by using motivational interviewing principles to facilitate person-centred change talk. Phases 2, 3, 4 and 6 utilise CBT principles to target maladaptive illness perceptions about CLBP and maladaptive coping strategies. In this way, the treatment phases are separated such that CBT principles are used to target maladaptive perceptions and coping behaviors, whereas motivational interviewing principles are used to target the desired adaptive behaviours. The lead author initially developed the contents of Table 5 and sent it to the research team. The final table was produced following a critical review and discussion by the research team.

**Table 5:** The six phases of each programme session

Phase 1	Education that is collaborative using motivational interviewing principles- obtaining permission from participants and ‘chunk-check-chunk’, that is give a little information at a time, check that this information is understood before giving another bit of information. This is followed by motivation building through engaging participants to describe their concerns and important life goals; and the barriers and facilitators to achieving them. Finally, there is agenda setting for the rest of the session.
Phase 2	Mapping of existing illness perceptions using CBT principles of assessment. Participants are stimulated to identify their illness beliefs and link the beliefs into CBT vicious cycle of beliefs/thoughts, mood, physical sensations, and behaviour.
Phase 3	Challenging maladaptive illness perceptions using CBT principles. Participants are encouraged to question their illness perceptions associated with maladaptive behaviour to explore if there is any utility (discovered by participants themselves) in having these perceptions.
Phase 4	Formulation of alternative illness perceptions and associated behaviours using CBT principles of guided discovery. Guided discovery involves participants being encouraged during group sessions to discover by themselves more positive ways of thinking and behaving in relation to their CLBP. Physiotherapist shows an understanding of participants’ point of view and encourages them to discover alternative ways of thinking about their concerns. Socratic dialogue is used to change maladaptive illness perceptions into alternative perceptions conducive to achieving life goals stated in phase 1.
Phase 5	Practising the alternative (desired) behaviour in a supervised session by completing the exercise and postural training sessions. After the cool down session, good posture in daily functional activities are practised. Participants are encouraged to explore alternative illness perceptions that may support performing exercises and adopting good posture in daily life. Subsequently, a plan for change is developed with the participants by exploring the incorporation of these activities into their daily lives. Motivational interviewing principles of ‘Elicit-provide-elicit’ are used. Physiotherapist facilitates the change talk by identifying and strengthening comments that show the desire to change, ability to change, reasons for change, need to change, commitment to change and taking steps towards behaviour change. Physiotherapist focuses at an individual level, on the most relevant area for each participant and stimulates the motivation to change by discussing outcome expectancies associated with exercises and good posture. For instance, participants can be told how exercising just before going to bed can improve pain and sleep, and how working from a table instead of the ground can reduce the number of pain episodes. There is exploration of participants’ risk awareness and self-efficacy. Goals to achieve the required behaviour change are set with the participants before the next session. Physiotherapist plans with the participants, the steps required for the exercises and good posture. Participants are encouraged to remember/record their activities. Social support is used to facilitate behaviour change by allowing each participant to identify a family member or significant other to support the exercises and good posture.
Phase 6	Testing of alternative illness perceptions and associated behaviours. Physiotherapist stimulates participants to test alternative illness perceptions and associated behaviours (exercises and good posture) by using CBT principles of behavioural experiments. Participants practise the behaviour (exercises and good posture) and appraise their efficacy in their lives. The desired behaviour is strengthened by participants confirming their utility in their daily life. This appears to correspond with the appraisal stage of the SRM. There is exploration of personal and social/environmental factors that may constitute barriers and facilitators to engaging in the exercises and good posture. Strategies to utilise the facilitators and reduce barriers to the desired change are explored with the participants. Culturally tailored goals are then set for personalised strategies to overcome the identified barriers. For instance, some participants who may not want to exercise on Sundays for spiritual reasons may be encouraged to exercise in the morning and later in the evening during the previous Saturday.

#### Physical and informational materials used in the intervention, and training of intervention provider

Patient booklet: This is an illustration-only booklet. Text was not included in the booklet due to low literacy rates in this rural population. Illustrations included culturally tailored exercise and good postural hygiene demonstrated using daily/work activities. Cultural tailoring involved using images of people that look like the community members, and including common activities performed in the rural community such as farming, and exercises that did not require special equipment in the booklet. The patient booklet will be published for programme implementation.

Physiotherapist booklet: This contains the content, rationale, and detailed procedure for program delivery. The booklet is designed as a clinical manual for delivering the program and a training manual for the delivering health professional. A clinical and training manual will be prepared for publication following a feasibility testing of programme implementation in the current Nigerian primary health care system. This would involve exploring the available health care personnel in primary care and the feasibility of training them to deliver this complex intervention.

Mats, chairs, tables, brooms, weights and hoes are used for performing exercises and demonstrating good postural hygiene.

#### Intervention provider, expertise, background and specific training

This was a doctoral level physiotherapist, with 15 years of clinical and research experience, who was supported by a multidisciplinary team comprising a physiotherapy professor and 2 senior academics in psychology and physiotherapy in developing the program. The intervention provider had received some training on CBT and motivational interviewing.

#### Intervention adherence and fidelity assessment

Program fidelity was assured by training the delivering health professional with the booklet. Video recordings of each program session was assessed independently by members of a multidisciplinary team. Assessments of health professional training and competence, adherence to intervention manual during delivery, intervention receipt, and the extent to which participants apply the self-management skills, will need to be quantified based on a scoring system in a subsequent study of the intervention. Participants’ adherence would need to be assessed in three domains: (1) number of program sessions attended; (2) to what extent participants adhered to recommended exercises; and good postural hygiene.

#### Modes of delivery

The program involves face-to-face interactive sessions supported by instruction manuals and graphic illustrations.

#### Location, necessary infrastructure or relevant features for intervention delivery

The program is designed for delivery in primary care with a room for the discussion sessions and a bigger room for the group exercise sessions.

#### Number of times for intervention delivery and period of time required: number of sessions, schedule, duration, intensity or dose

Six sessions delivered once weekly over 6 weeks. Each session lasts for 2 hours with an additional thirty-minute break period for refreshment. Each session consists of the educational component (phase 1) that lasts for 30 minutes. This is followed by a break period of 15 minutes. Phases two, three and four last for 45 minutes. Phase five lasts for 45 minutes, followed by another fifteen-minute break. Phase six is the final stage and lasts for 20 min.

#### Tailoring and adapting the intervention: what, why, when and how

Behavioral experiments, behavior-change goals and action plans can be individualised and tailored to suit individual participants. Tailoring is based on individual life goals and identified barriers and facilitators to behavior change, highlighted during the interactive group discussions.

## Results

### Initial program implementation and feasibility testing

The initial feasibility and acceptability of the program was investigated using an exploratory pragmatic non-randomised controlled study incorporating qualitative individual exit feedback interviews [45]. This study took place in a rural primary care centre serving about 15,000 rural dwellers in Enugu state of South-eastern, Nigeria. The program was delivered by a highly trained specialised physiotherapist, once weekly. The physiotherapist had Bachelors, Masters and PhD level qualifications; several training courses on CBT and motivational interviewing; plus several years of clinical experience in chronic disease management. Inclusion criteria include participants aged 18 years and above, with LBP lasting for over 12 weeks. Those with non-specific CLBP were identified by screening to exclude any underlying serious pathology such as fractures, malignancy, infection, inflammation, and radicular syndrome. Thirteen participants received the Good Back program and nine participants received usual care (control group) for 6 weeks. The usual care group did not receive any intervention and were allowed to take up treatments they would normally use without interference since the aim of the study was to compare the feasibility and outcome of the program when compared with usual care [45]. Based on previous evidence, usual care in this population could comprise analgesic drugs including opioids obtained from patent medicine sellers usually without prescription, herbal remedies from herbalists, and spiritual healing attempts from spiritual healers [18, 19]. Convenient assignment into both arms was done within the second week of November 2015, and ensured that the few interested younger adults, male participants and non-farmers could be assigned equally into the two groups, and that non-participation could be reduced in this very small sample. Pre- and post-test outcome measurements were performed by a trained physiotherapist unaware of group assignment. Post treatment assessment was done 2 days after the last program session. All measures except performance-based disability and blood pressure were self-reported, and therefore were interviewer-administered using cross culturally adapted measures.

Recruitment rate was 100%, program attendance was 83%, loss to follow up/dropout rate was 8% compared to 11% in the usual care/control group. Table 6, copied from Igwesi-Chidobe et al. [45], shows that the primary outcome of self-reported disability (Roland Morris Disability Questionnaire – RMDQ), and secondary outcomes of fear avoidance beliefs (fear avoidance beliefs questionnaire – FABQ), systolic and diastolic blood pressure were balanced in the two groups. Greater benefits for the self-management group compared with control were observed for primary and secondary biopsychosocial outcomes including self-reported disability measured with the Roland Morris Disability Questionnaire – RMDQ [− 9.8 (4.8) versus 1.5 (3.9)]; performance based disability measured with the Back Performance Scale – BPS [− 0.8 (1.3) versus 0.3 (2.8)]; illness perceptions measured with the Brief Illness Perceptions Questionnaire – BIPQ [− 21.6 (15.2) versus 3.8 (7.5)]; fear avoidance beliefs measured with the fear avoidance beliefs questionnaire – FABQ [− 41.7 (27.6) versus 7.8 (35.8)]; pain intensity measured with the 11-point box scale – 11-BS [− 3.8 (2.1) versus 1.5 (3.1)]; and pain medication use [− 14.6 (12.1) versus − 2.8 (20.3)] (Table 6). Male participants had erratic program attendance with none attending all sessions, and had less improved outcomes for self-reported disability, illness perceptions, fear avoidance beliefs and pain intensity when compared with female participants. Qualitative inductive content analysis was performed. Interview transcripts were first translated to English. English interview transcripts were then organised into thematic areas, and the number of people reporting each theme and subtheme were noted using NVivo version 10. The analysis utilised the manifest content of the transcripts due to the structured format of the interviews that directly answered specific questions for programme improvement [45]. Inductive content analysis of interviews showed that the program was acceptable to participants. However, they made recommendations for program improvement which included shorter but ongoing sessions incorporating video demonstration of exercises and good posture, the use of primary care centres with spacious exercise/demonstration rooms, and changing negative community beliefs about exercises to legitimise exercise as treatment for back pain thereby reducing the current stigma associated with it [45].

**Table 6:** Baseline demographic and clinical characteristics; and means and effect sizes in the self-management and usual care (control) groups (copied from [45])

Variables	**BASELINE DEMOGRAPHIC AND CLINICAL CHARACTERISTICS IN THE TWO STUDY ARMS**								
	Good Back programme *n* = 13				Usual care (control) *n* = 9				
Gender: frequency (%)									
Female	10 (76.9)				6 (66.7)				
Male	3 (23.1)				3 (33.3)				
Age (years): mean (SD)	53.9 (14.1)				60.3 (13.6)				
Education (years): mean (SD)	5.3 (5.4)				4.4 (4.7)				
Marital status: frequency (%)									
Married	7 (53.8)				5 (55.6)				
Widowed	5 (38.5)				4 (44.4)				
Single	1 (7.7)				0 (0)				
Occupation: frequency (%)									
Self-employed/trading/farming	8 (61.5)				6 (66.7)				
Unemployed (health reasons)	3 (23.1)				2 (22.2)				
Student	1 (7.7)				0 (0)				
Retired	1 (7.7)				1 (11.1)				
Religion: frequency (%)									
Catholic	7 (53.8)				6 (66.7)				
Anglican	3 (23.1)				0 (0)				
Methodist	2 (15.4)				0 (0)				
Pentecostal	1 (7.7)				3 (33.3)				
Co-morbidity: frequency (%)									
None	5 (38.5)				1 (11.1)				
HBP	5 (38.5)				6 (66.7)				
Diabetes	1 (7.7)				0 (0)				
Headache+toothache	1 (7.7)				1 (11.1)				
Eye problems	1 (7.7)				1 (11.1)				
LBP duration (years): mean (SD)	6.8 (4.1)				9.3 (15.4)				
Systolic blood pressure (mmHg): mean (SD)	130.5 (23.5)				130.0 (32.2)				
Diastolic blood pressure (mmHg): mean (SD)	80.7 (9.5)				75.0 (10.5)				
Pain tablets in the last 2 weeks: mean (SD)	17.3 (14.0)				28.2 (34.2)				
RMDQ: mean (SD)	17.0 (5.8)				17.8 (4.6)				
Pain intensity (Igbo-11-BS): mean (SD)	6.8 (1.7)				5.3 (1.6)				
BIPQ: mean (SD)	34.9 (10.7)				41.1 (9.7)				
FABQ: mean (SD)	64.0 (21.9)				65.3 (15.8)				
BPS: mean (SD)	3.2 (2.1)				4.6 (2.7)				
	**POST-INTERVENTION MEANS AND EFFECT SIZES IN THE TWO STUDY ARMS**								
	Post-test mean values (SD)		Within-group/Pre- and post-test mean differences (SD)		95% Confidence intervals of within-group mean differences		Between-group mean differences	95% Confidence intervals of between-group differences	Between-group effect sizes Hedges’ g (Glass’s Δ)
	Good back programme *n* = 12	Usual care (waiting list) *n* = 8	Good back programme *n* = 12	Usual care (waiting list) *n* = 8	Good back programme *n* = 12	Usual care (waiting list) *n* = 8			
RMDQ	7.0 (4.2)	19.6 (5.6)	−9.8 (4.8)	1.5 (3.9)	−12.8, −6.7	−1.8, 4.8	−12.6	− 17.2, −8.0	−2.5 (−2.2)
BPS	2.1 (1.6)	4.6 (2.7)	−0.8 (1.3)	0.3 (2.8)	−1.6, − 0.03	−2.1, 2.6	−2.5	−4.5, − 0.5	−1.2 (− 1.0)
BIPQ	13.5 (9.5)	45.6 (15.1)	− 21.6 (15.2)	3.8 (7.5)	−31.3, −11.9	−2.5, 10.0	−32.1	−43.6, − 20.6	−2.6 (− 2.1)
FABQ	20.3 (15.9)	73.5 (28.5)	− 41.7 (27.6)	7.8 (35.8)	− 59.2, − 24.1	−22.2, 37.7	−53.2	−74.0, − 32.4	−2.3 (− 1.9)
11-BS	2.8 (1.3)	7.0 (2.5)	−3.8 (2.1)	1.5 (3.1)	−5.2, −2.5	−1.1, 4.1	−4.2	−5.9, − 2.4	−2.2 (− 1.7)
Number of Pain tablets	3.2 (5.0)	28.5 (37.8)	− 14.6 (12.1)	−2.8 (20.3)	− 22.3, − 6.9	−19.7, 14.2	−25.3	−48.3, − 2.4	−1.0 (− 0.7)
SBP (mmHg)	118.3 (11.6)	131.3 (30.0)	−12.5 (23.7)	2.0 (35.6)	−29.5, 4.5	−42.2, 46.2	− 13.0	−32.9, 6.9	−0.6 (− 0.4)
DBP (mmHg)	70.8 (13.3)	75.6 (15.0)	−5.8 (14.4)	0.0 (10.0)	−16.1, 4.5	−12.4, 12.4	−4.8	− 18.2, 8.6	−0.3 (− 0.3)

*SD* standard deviation

### Refined good Back program: changes made following feasibility testing

The refined program is presented in Table 7 below. The changes made to the program are enclosed in orange boxes. In response to study participants’ recommendations, the minor modifications below (in bold) were made to the program. The research team, steered by the lead author, met on two occasions to discuss the findings from the feasibility study [45] and how the initial program can be modified incorporating these. The lead author modified Table 3 incorporating these suggestions to produce Table 7.

1. Program structure and delivery: the program may need to be ongoing or have booster sessions and be timed to end by one pm before primary school dismissal to allow individuals to collect their children from school. There should be inclusion of videos of the models practicing the exercises and good posture demonstrated during the program sessions. Every participant in the self-management program should be given a video and a patient booklet at the start of the program.
2. Primary health care facility: a bigger room or an open space that will allow free movement during the exercise and postural training sessions is required to deliver the program.
3. Changes at the community level: it may be important to try to influence the rural communities’ beliefs and attitudes towards exercises and good postural hygiene as management strategies for CLBP in order to support exercise-related behaviour change, especially when the program sessions are over. People were mocked for adopting exercises as a treatment strategy during the program sessions in this community that regarded only pharmacological treatment as legitimate as highlighted by one participant’s comment *‘… they were saying, “where are the drugs you were given for your pain”? Then they laughed and said, “you are just going to the health centre to play” … ‘(P10)* [45]. Therefore, the participants suggested the use of mass media to create more awareness about behavioural management of CLBP such as exercise. Consequently, behavioural journalism, a social cognitive theory related method [24], is included in response to recommendations for using mass media to inform the community about the legitimacy of exercises for managing CLBP. Behavioral journalism will be incorporated via community-wide exercise campaigns with real life role model stories of people for whom exercise improved their CLBP. This will be the first aspect of the intervention and will be done prior to commencing the program sessions in the health centres assigned to the intervention group. This is expected to reduce the resistance participants may face from the community in response to the long-term adoption of exercise for managing their CLBP, thereby supporting long-term behaviour change. This concurs with the systematic review finding that social environmental modification supports physical activity behaviour change [26].

**Table 7:**
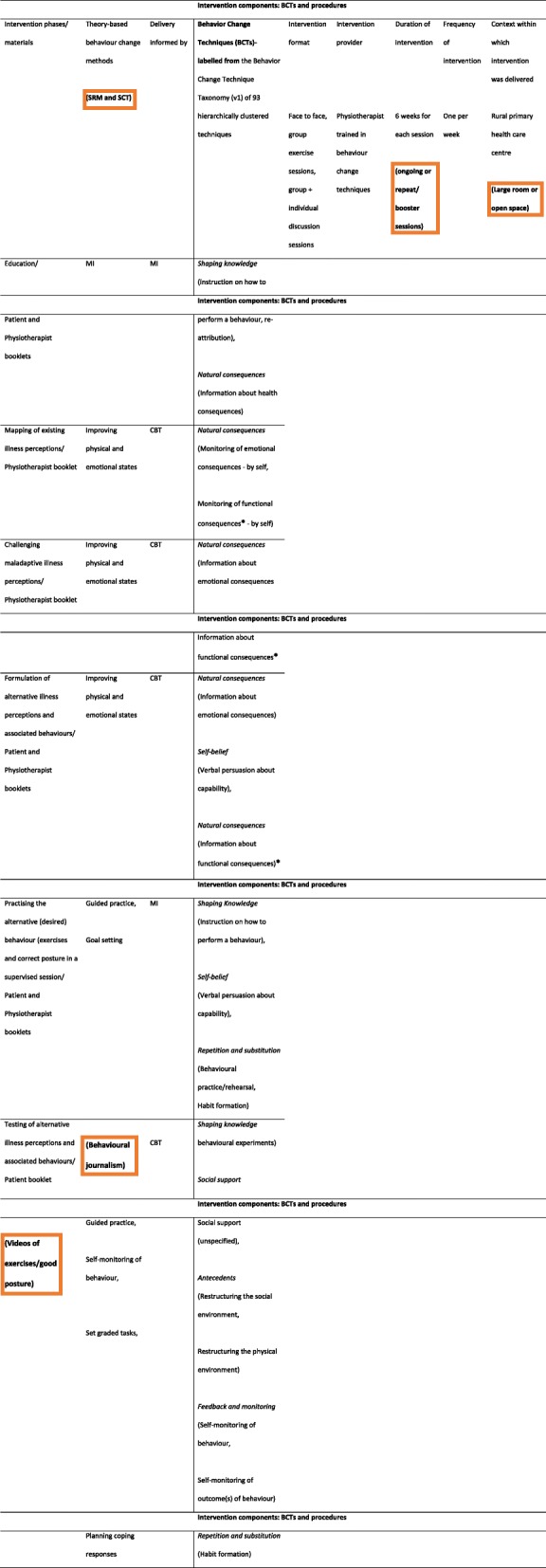
The refined Good Back programme

*SRM* Leventhal’s self-regulatory model of illness cognitions, *SCT* Bandura’s Social Cognitive Theory, *MI* Motivational interviewing, *CBT* Cognitive behavioural therapy, * absent in the 93 BCTs,  modifications made to the programme

### Theoretical underpinning of the refined good Back program

The refined program is underpinned by the self-regulatory model (SRM) of illness cognitions [27] and social cognitive theory (SCT) [46].

#### Self-regulatory model (SRM) of illness cognitions

**Fig. 2:**
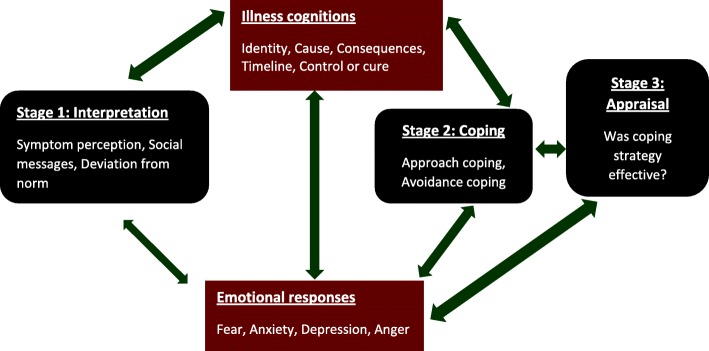
Leventhal’s self-regulatory model of illness cognitions [adapted from the lead author’s PhD thesis^**17**^]

According to the SRM, an individual confronts a potential illness via symptom perception and social messages (Fig. 2).

The individual then assigns a meaning to the problem, expressed as particular beliefs or illness cognitions, which are presented in five domains. “Identity” is the individual’s understanding and label of the illness and the associated symptoms. “Cause” is personal beliefs about the cause of the illness. “Timeline” illustrates how long the individual believes the illness will last. “Control/cure” is the extent to which the individual believes s/he can control or recover from the illness. “Consequences” are the individual’s beliefs about the outcomes and effects of the condition on his/her life [27]. Illness cognitions are also known as illness beliefs, illness perceptions or illness representations.

Further research has resulted in the addition of “illness coherence” (how well a patient feels they understand the illness), “treatment control” (how much treatment is believed to help/control the illness), “personal control” and “emotional representation” (how much patients are emotionally affected by the illness). Emotional representations are expressed concurrently with cognitions, in response to symptom perception and social messages. Emotional representations affect illness cognitions and both influence symptom interpretation in a bidirectional relationship (Fig. 2).

Due to the motivation to return to a problem-free state, the individual’s illness cognitions influence any health-related behaviours and coping strategies adopted. Adopted coping strategies then impact on disease outcomes. Finally, during appraisal, the individual evaluates the coping strategies used as either effective or ineffective. The coping strategy will be continued if appraised as effective and discontinued if seen as ineffective. In the latter, the individual will be motivated to search for alternative strategies. Importantly, emotional representations also affect coping strategies and appraisal of coping strategies. The model is known as self-regulatory because the three steps are inter-related and ongoing in a dynamic process [27].

#### Social cognitive theory (SCT)

The theoretical modification of the program was the addition of the SCT, informed by the results of this study.

**Fig. 3:**
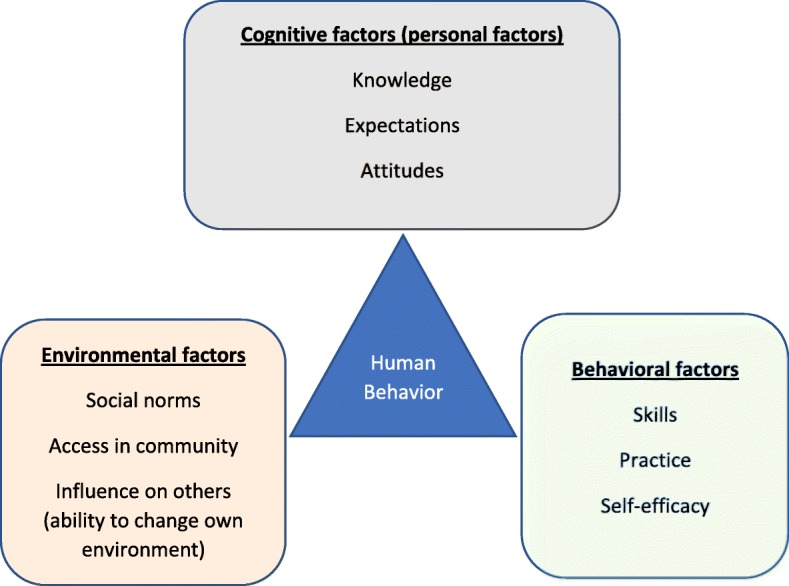
Bandura’s Social Cognitive Theory [adapted from the lead author’s PhD thesis^**17**^]

The SCT explains that learning is affected by cognitive, behavioural, and environmental factors in a reciprocal relationship [46]. The SCT specifies five core sets of determinants of health behaviour: knowledge, perceived self-efficacy, outcome expectations, goals and perceived facilitators/environmental impediments (Fig. 3).

Knowledge is the starting point and a precondition for behaviour change and was acknowledged in developing the program. People will only want to change their behaviour if they have a knowledge about how their lifestyle affects their health. However, knowledge is insufficient for behaviour change, and personal beliefs about the ability to enact the behaviour (perceived self-efficacy) are required to overcome barriers associated with changing and maintaining behaviour. Whether an individual adopts a healthy behaviour is also determined by the outcomes they expect from the behaviour (outcome expectations). Outcome expectations include physical outcomes, including losses and benefits, social outcomes such as the approval or disapproval the behaviour brings about in relationships to others, and personal outcomes which concerns the positive or negative self-evaluative reactions to the health behaviour and associated health outcomes. Goals include personal aspirations that people value highly. Short term goals help people to change behaviour by guiding their actions in the here and now, and are more influential than long term goals which may be associated with competing interests. Perceived facilitators and barriers, which include personal and environmental factors, also determine health behaviour. Individuals are more likely to adopt a health behaviour when they perceive fewer personal and societal impediments to that behaviour [46].

Of the five core determinants of behaviour in the SCT, perceived self-efficacy is the most fundamental as it affects health behaviour both directly and indirectly through its effects on the other determinants. People with stronger perceived self-efficacy, set higher goals and are more committed to achieving them, expect more favourable outcomes, view barriers as surmountable by the improvement of self-management skills, and are more likely to persevere in the presence of obstacles [46]. Self-efficacy was acknowledged in the development of the Good Back program by targeting people’s beliefs in their ability to formulate adaptive illness cognitions about CLBP, and practice exercises and good posture for managing it.

Communities’ beliefs and attitudes towards exercising and adopting good posture for managing CLBP, labelled as social outcomes (outcome expectations) in the SCT [46], were not targeted by the Good Back program initially as it was not covered by the SRM. This is now included as this was suggested by participants to be a salient factor that may influence exercise adoption and maintenance in rural Nigeria.

#### Integration of the self-regulatory model (SRM) of illness cognitions and social cognitive theory (SCT)

**Fig. 4:**
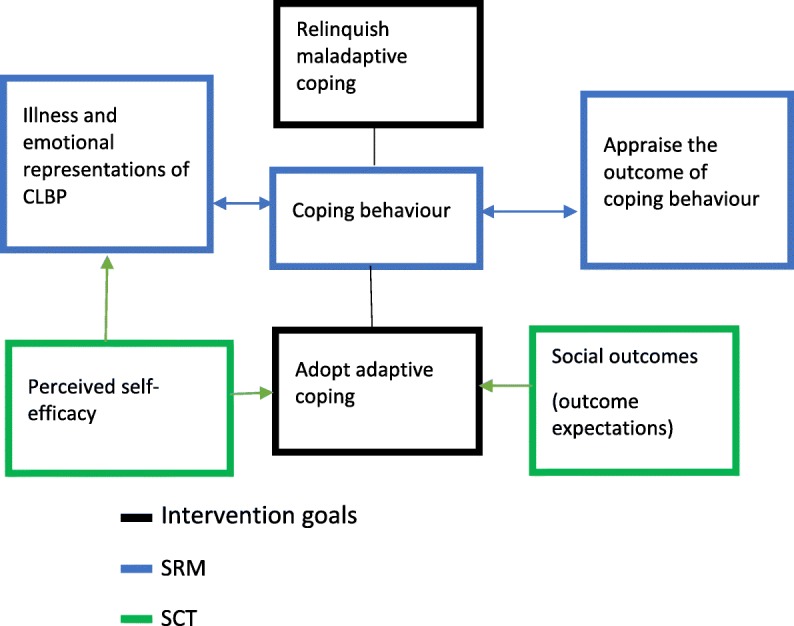
[adapted from the lead author’s PhD thesis^**17**^]**:** Theoretical underpinning of refined Good Back program involving the Leventhal’s self-regulatory model (SRM) of illness cognitions and aspects of Bandura’s social cognitive theory (SCT)

Figure 4 below summarises the theoretical underpinning of the refined program by the integration of the SRM and the SCT.

The aspects of the refined intervention that target or challenge maladaptive back pain beliefs such as cure seeking, and maladaptive coping strategies such as drug dependence, are underpinned by the SRM; whereas parts of the refined program focusing on the adoption of adaptive coping strategies such as exercise and good postural hygiene are underpinned by the SCT (Fig. 4).

## Discussion

This paper discusses the development of a complex biopsychosocial behaviour change intervention for adults living with non-specific CLBP in rural Nigeria – the Good Back Program. The feasibility of conducting a randomised controlled trial (RCT) of the Good Back program could not be determined as there were too many unknowns in this under-studied population with a predominant acute-infective-pharmacological primary health care model. Therefore, the focus of an initial study was to determine the feasibility and acceptability of this non-pharmacological behaviour-change intervention for CLBP – a chronic non-communicable condition, for the first time in rural Nigerian primary health care. Evidence-based guidelines support conducting RCTs of implementable interventions to enable their adoption into routine health care if found effective [47]. Hence, it is important to consider the feasibility of implementing the Good Back Program within the current Nigerian primary health care structure alongside or prior to investigating the feasibility of a RCT.

As the program is a complex intervention, future studies need to test the feasibility and acceptability of training frontline Nigerian primary health care staff with less professional training than physiotherapists (who do not currently practice in Nigerian primary care) to deliver the intervention. Evidence suggests that complex interventions can be delivered by people with minimal or no qualifications in African settings. For instance, a complex CBT-based mental health program was successfully delivered by lay health workers in primary health care centres in Zimbabwe [48]. The lay workers were trained, used detailed script contained in a manual, and were supervised by a health supervisor trained in mental health [48]. Community health workers, who are the frontline of rural Nigerian primary health care, and who have 2–3 years post-secondary education that enables analytic and assessment skills, programme planning skills, communication skills, cultural competency skills, community dimension skills, public health science skills, financial/management skills and leadership thinking skills, might be able to deliver the Good Back Program. Their training needs and possible supervision by competent health professionals such as community physiotherapists need to be explored for program implementation in a subsequent feasibility study. This supports the 2014 Nigerian ministry of health policy recommendation of task-shifting of essential primary health care services from more specialised health professionals unavailable in rural primary health care, to adequately trained community health workers [49].

As a behavior change intervention, the Good Back Program facilitated the relinquishment of maladaptive behaviors and adoption of adaptive behaviors. Dependence on analgesic drugs (including opiates), which is a maladaptive coping behaviour, was measured by counting the number of pain medications taken in the last 2 weeks. This measurement assumes that the number of tablets is proportional to the dosage which is not always the case. Measurement of dose in specific units such as grams or milligrams would have provided a more accurate measurement, but this would have been impossible in this rural population with low literacy rates. Despite the apparent limitation of counting the number of tablets, it enabled some rough estimation of the amount of pain medications being taken in this population. The adaptive behaviors included adoption of regular exercise and good postural hygiene during daily functional activities. Exercise adoption was measured with the exercise adherence rating scale (EARS), a self-report measure with very good psychometric properties [50]. Adoption of good postural hygiene was not assessed in the initial feasibility due to a lack of an adequate measure for assessing it. Presently, no measure exists to assess the adoption of good postural hygiene during functional activities. Therefore, a new measure may need to be developed for a future RCT in rural Nigeria to enable measured outcomes to reflect the theoretical constructs of the programme and clarify the mechanisms through which outcomes are produced. Similarly, a new measure may need to be developed to better reflect the pain coping strategies commonly adopted in rural Nigeria, as existing pain coping strategies measures do not appear to be suitable for this population.

The IM approach has proven useful in the development of a complex behavior change intervention in rural Nigeria. The approach enabled the use of theory-based methods and BCTs in intervention development. Self-monitoring, which is encouraged by asking participants to keep a record of their exercises and functional activities, has been found to be one of the most important BCTs for improving physical activity in both high income [51] and low income countries including those in Africa [26]. However, two BCTs labelled as monitoring of functional consequences (participants asked to explore how their beliefs, thoughts and behavior influenced their daily functional activities), and information about functional consequences (strategies used to improve daily functional activities) were not in the current taxonomy of 93 BCTs which included emotional consequences and no functional consequences [30]. The lack of representation of functional consequences could be because this taxonomy has been developed predominantly from a psychological point of view. This has important implications for the future development of the taxonomy.

### Strengths and limitations

IM appears very useful in developing evidence-based theory-informed interventions suitable for rural African contexts. However, the approach is complex, and requires that considerable research is carried out before developing a new intervention. Nonetheless, the approach may make it easier to adapt complex interventions for different contexts, particularly those in low-income settings.

### Implications for theory, policy and practice

The initial feasibility testing of this intervention appears promising [45] but further testing of the program is required. A rigorous testing in a randomised controlled trial would allow an investigation of clinical and cost effectiveness, a further exploration of specific theories, and how specific theoretical constructs may drive back pain behavior in this population.

Another important consideration is who should deliver this program to enable implementation. For the initial development, the program was physiotherapist-led. However, it might be more feasible to train community health workers, who are the front-line primary health care workers in rural Nigeria, to deliver the intervention, supervised by community physiotherapists.

## Conclusions

The IM approach has proven useful in the development and initial feasibility testing of a community-based self-management program for non-specific CLBP in a rural African primary care centre. This approach might be useful in developing other complex behaviour change interventions for chronic conditions in rural African contexts.

## Data Availability

Not applicable. Published/Secondary data were used in this study and were referenced. All data can be obtained from the referenced studies or from the lead author.

## Abbreviations

BCTs: Behavior Change Techniques
CBT: Cognitive Behavioral Therapy
CLBP: Chronic Low Back Pain
IM: Intervention Mapping
LBP: Low Back Pain
SCT: Social Cognitive Theory
SRM: Self-Regulatory Model of illness perceptions

## References

[1] Vos T, Barber RM, Bell B, Bertozzi-Villa A, Biryukov S, Bolliger I, Charlson F, Davis A, Degenhardt L, Dicker D, Duan L (2015). Global, regional, and national incidence, prevalence, and years lived with disability for 301 acute and chronic diseases and injuries in 188 countries, 1990–2013: a systematic analysis for the global burden of disease study 2013. Lancet.

[2] Clark S, Horton R (2018). Low back pain: a major global challenge. Lancet.

[3] Hoy D, Brooks P, Blyth F, Buchbinder R (2010). The epidemiology of low back pain. Best Pract Res Clin Rheumatol.

[4] Hartvigsen J, Hancock MJ, Kongsted A, Louw Q, Ferreira ML, Genevay S, Hoy D, Karppinen J, Pransky G, Sieper J, Smeets RJ (2018). What low back pain is and why we need to pay attention. Lancet.

[5] Hoy D, Toole MJ, Morgan D, Morgan C (2003). Low back pain in rural Tibet. Lancet.

[6] Igwesi-Chidobe CN, Coker B, Onwasigwe CN, Sorinola IO, Godfrey EL (2017). Biopsychosocial factors associated with chronic low back pain disability in rural Nigeria: a population-based cross-sectional study. BMJ Glob Health.

[7] Omokhodion FO (2004). Low back pain in an urban population in Southwest Nigeria. Trop Dr.

[8] Tella B, Akinbo S, Asafa S, Gbiri C (2013). Prevalence and impacts of low back pain among peasant farmers in south-West Nigeria. Int J Occup Med Environ Health.

[9] Foster NE, Anema JR, Cherkin D, Chou R, Cohen SP, Gross DP, Ferreira PH, Fritz JM, Koes BW, Peul W, Turner JA (2018). Prevention and treatment of low back pain: evidence, challenges, and promising directions. Lancet.

[10] Michie S, Van Stralen MM, West R (2011). The behaviour change wheel: a new method for characterising and designing behaviour change interventions. Implement Sci.

[11] Dolan P, Hallsworth M, Halpern D, King D, Metcalfe R, Vlaev I (2012). Influencing behaviour: the mindspace way. J Econ Psychol.

[12] Eldredge LK, Markham CM, Ruiter RA, Kok G, Fernandez ME, Parcel GS (2016). Planning health promotion programs: an intervention mapping approach.

[13] Craig P, Dieppe P, Macintyre S, Michie S, Nazareth I, Petticrew M (2013). Developing and evaluating complex interventions: the new Medical Research Council guidance. Int J Nurs Stud.

[14] Draper CE, Micklesfield LK, Kahn K, Tollman SM, Pettifor JM, Dunger DB, Norris SA (2014). Application of intervention mapping to develop a community-based health promotion pre-pregnancy intervention for adolescent girls in rural South Africa: project Ntshembo (Hope). BMC Public Health.

[15] Aarø LE, Flisher AJ, Kaaya S, Onya H, Fuglesang M, Klepp KI, Schaalma H (2006). Promoting sexual and reproductive health in early adolescence in South Africa and Tanzania: development of a theory-and evidence-based intervention programme. Scand J Public Health.

[16] Hoffmann TC, Glasziou PP, Boutron I, Milne R, Perera R, Moher D, Altman DG, Barbour V, Macdonald H, Johnston M (2014). Better reporting of interventions: template for intervention description and replication (TIDieR) checklist and guide. BMJ.

[17] Igwesi-Chidobe CN. Development and preliminary evaluation of a self-management programme for people with non-specific chronic low back pain in rural Nigeria (doctoral dissertation, King’s College London). 2017.

[18] Igwesi-Chidobe CN, Kitchen S, Sorinola IO, Godfrey EL (2017). “A life of living death”: the experiences of people living with chronic low back pain in rural Nigeria. Disabil Rehabil.

[19] Igwesi-Chidobe CN, Sorinola IO, Kitchen S, Godfrey EL (2018). Unconventional practitioners’ causal beliefs and treatment strategies for chronic low back pain in rural Nigeria. Health Serv Insights.

[20] Igwesi-Chidobe CN, Obiekwe C, Sorinola IO, Godfrey EL. Assessing self-reported disability in a low-literate population with chronic low back pain: cross-cultural adaptation and psychometric testing of Igbo Roland Morris disability questionnaire. Disabil Rehabil. 2017. 10.1080/09638288.2017.1416185. 10.1080/09638288.2017.1416185 29239235

[21] Kamper SJ, Apeldoorn AT, Chiarotto A, Smeets RJ, Ostelo RW, Guzman J, Van Tulder MW (2015). Multidisciplinary biopsychosocial rehabilitation for chronic low back pain: Cochrane systematic review and meta-analysis. BMJ.

[22] Tsauo JY, Chen WH, Liang HW, Jang Y (2009). The effectiveness of a functional training programme for patients with chronic low back pain–a pilot study. Disabil Rehabil.

[23] Schenkman ML, Jordan S, Akuthota V, Roman M, Kohrt WM, Hearty T, Cleary C, Backstrom KM (2009). Functional movement training for recurrent low back pain: lessons from a pilot randomized controlled trial. PM&R..

[24] Kok G, Gottlieb NH, Peters GJ, Mullen PD, Parcel GS, Ruiter RA, Fernández ME, Markham C, Bartholomew LK (2016). A taxonomy of behaviour change methods: an intervention mapping approach. Health Psychol Rev.

[25] Rhodes RE, de Bruijn GJ (2013). How big is the physical activity intention–behaviour gap? A meta-analysis using the action control framework. Br J Health Psychol.

[26] Igwesi-Chidobe CN, Kengne AP, Sorinola IO, Godfrey EL (2018). Physical activity containing behavioural interventions for adults living with modifiable chronic non-communicable diseases in Africa: a systematic mixed-studies review. Int Health.

[27] Leventhal H, Leventhal EA, Contrada RJ (1998). Self-regulation, health, and behavior: a perceptual-cognitive approach. Psychol Health.

[28] Foster NE, Thomas E, Bishop A, Dunn KM, Main CJ (2010). Distinctiveness of psychological obstacles to recovery in low back pain patients in primary care. Pain.

[29] Siemonsma PC, Stuive I, Roorda LD, Vollebregt JA, Walker MF, Lankhorst GJ, Lettinga AT (2013). Cognitive treatment of illness perceptions in patients with chronic low back pain: a randomized controlled trial. Phys Ther.

[30] Michie S, Richardson M, Johnston M, Abraham C, Francis J, Hardeman W, Eccles MP, Cane J, Wood CE (2013). The behavior change technique taxonomy (v1) of 93 hierarchically clustered techniques: building an international consensus for the reporting of behavior change interventions. Ann Behav Med.

[31] Henschke N, Ostelo RW, van Tulder MW, Vlaeyen JW, Morley S, Assendelft WJ, Main CJ (2010). Behavioural treatment for chronic low-back pain. Cochrane Database Syst Rev.

[32] Williams AC, Eccleston C, Morley S (2012). Psychological therapies for the management of chronic pain (excluding headache) in adults. Cochrane Database Syst Rev.

[33] Jonsbu E, Martinsen EW, Morken G, Moum T, Dammen T (2013). Change and impact of illness perceptions among patients with non-cardiac chest pain or benign palpitations following three sessions of CBT. Behav Cogn Psychother.

[34] Dobson D, Dobson KS (2018). Evidence-based practice of cognitive-behavioral therapy.

[35] Linehan MM (2018). Cognitive-behavioral treatment of borderline personality disorder.

[36] Mann E, Smith MJ, Hellier J, Balabanovic JA, Hamed H, Grunfeld EA, Hunter MS (2012). Cognitive behavioural treatment for women who have menopausal symptoms after breast cancer treatment (MENOS 1): a randomised controlled trial. Lancet Oncol.

[37] Siddons HM, Wootten AC, Costello AJ (2013). A randomised, wait-list controlled trial: evaluation of a cognitive–behavioural group intervention on psycho-sexual adjustment for men with localised prostate cancer. Psycho-Oncology..

[38] Otis JD, Sanderson K, Hardway C, Pincus M, Tun C, Soumekh S (2013). A randomized controlled pilot study of a cognitive-behavioral therapy approach for painful diabetic peripheral neuropathy. J Pain.

[39] Rodriguez-Hernandez H, Morales-Amaya UA, Rosales-Valdéz R, Rivera-Hinojosa F, Rodriguez-Moran M, Guerrero-Romero F (2009). Adding cognitive behavioural treatment to either low-carbohydrate or low-fat diets: differential short-term effects. Br J Nutr.

[40] Barrowclough C, Haddock G, Wykes T, Beardmore R, Conrod P, Craig T, Davies L, Dunn G, Eisner E, Lewis S, Moring J (2010). Integrated motivational interviewing and cognitive behavioural therapy for people with psychosis and comorbid substance misuse: randomised controlled trial. BMJ.

[41] Jones SH, Barrowclough C, Allott R, Day C, Earnshaw P, Wilson I (2011). Integrated motivational interviewing and cognitive–behavioural therapy for bipolar disorder with comorbid substance use. Clin Psychol Psychother.

[42] Miller WR, Rollnick S (2012). Motivational interviewing: helping people change.

[43] Ehde DM, Dillworth TM, Turner JA (2014). Cognitive-behavioral therapy for individuals with chronic pain: efficacy, innovations, and directions for research. Am Psychol.

[44] Lawn S, Schoo A (2010). Supporting self-management of chronic health conditions: common approaches. Patient Educ Couns.

[45] Igwesi-Chidobe CN, Godfrey EL, Kitchen S, Onwasigwe CN, Sorinola IO (2019). Community-based self-management of chronic low back pain in a rural African primary care setting: a feasibility study. Prim Health Care Res Dev.

[46] Bandura A (2011). Social cognitive theory. Handbook of social psychological theories.

[47] Moore GF, Audrey S, Barker M, Bond L, Bonell C, Hardeman W, Moore L, O’Cathain A, Tinati T, Wight D, Baird J (2015). Process evaluation of complex interventions: Medical Research Council guidance. BMJ..

[48] Chibanda D, Weiss HA, Verhey R, Simms V, Munjoma R, Rusakaniko S, Chingono A, Munetsi E, Bere T, Manda E, Abas M (2016). Effect of a primary care–based psychological intervention on symptoms of common mental disorders in Zimbabwe: a randomized clinical trial. JAMA..

[49] Federal Ministry of Health. Task-shifting and task-sharing policy for essential health care services in Nigeria: Federal Ministry of Health; 2014. https://advancefamilyplanning.org/sites/default/files/resources/Nigeria%20taskshifting%20policy-Aug2014%20REVISEDCLEAN%20_Approved%20October%202014.pdf.

[50] Newman-Beinart NA, Norton S, Dowling D, Gavriloff D, Vari C, Weinman JA, Godfrey EL (2017). The development and initial psychometric evaluation of a measure assessing adherence to prescribed exercise: the exercise adherence rating scale (EARS). Physiotherapy..

[51] Michie S, Abraham C, Whittington C, McAteer J, Gupta S (2009). Effective techniques in healthy eating and physical activity interventions: a meta-regression. Health Psychol.

